# Pharmacy Education Development

**DOI:** 10.3390/pharmacy9040168

**Published:** 2021-10-13

**Authors:** Claire Anderson, Naoko Arakawa

**Affiliations:** Division of Pharmacy Practice and Policy, School of Pharmacy, University of Nottingham, Nottingham NG7 2RD, UK; Naoko.Arakawa@nottingham.ac.uk

Pharmacy education and training is fundamental in supplying the pharmacy workforce with adequate numbers and correct competencies to provide relevant pharmaceutical care. Many different countries are at various levels of development with undergraduate and postgraduate pharmacy education due to their economy, infrastructure, or their academic capacity. Pharmacy education development is multifaceted research and practice to ultimately optimize and improve patients’ and public health. This can include establishing a pharmacy education program itself; developing and assisting institutions function to be sustainable and evidence-based student-centered learning communities; and enhancing curriculum design, teaching, and learning to develop high-quality student learning experiences. The wide variety of initial/preservice and postgraduate pharmacy education development all over the world stimulates learning between countries, regions, and individual universities according to their needs.

This Special Issue brings together 17 original articles related to pharmacy education development at individual university, local/regional, country, and international levels. Three trends stand out: delivery techniques, delivering content relevant to the real word, and ensuring the quality of the education that we deliver. The pandemic has accelerated curricular change, and many of the papers in this Special Issue describe and begin to evaluate those changes.

The pandemic accelerated a move to digital and blended teaching and learning. While many institutions were already delivering digital teaching and learning, academics across the globe had to quickly learn new skills and work long hours to quickly move their courses to digital platforms. As Mirzaian and Franson write, digital transformation in any industry is not a simple undertaking, but when the pandemic wanes and educational institutions teach face-to-face again, they will be able to embrace some of the changes made during the pandemic and make a more strategic shift to transform education [1].

Mak and colleagues in Australia changed performance-based assessments using patient simulation to examine students’ competence in clinical knowledge and communication skills to a digital exercise [2]. Adaptations to reduce reliance on internet access were made where students submitted a video-recording task, wherein they educated a simulated patient on a medicines list under lockdown restrictions during the pandemic. Students performed role-play with a simulated patient, such as their family members, housemates, or peers, either at home, in person, or via Zoom^TM^. The task increased the awareness of patient literacy, utilizing medicines lists for medication self-management. The authors state that, “pharmacy education in particular, has witnessed a remarkable shift during this unprecedented time, creating meaningful and relevant opportunities for students to prepare themselves for the imminent digitalization of healthcare. Introducing students to simulation activities via telehealth, to increase health literacy, is clearly a silver lining in this extraordinary circumstance.”

The utilization of serious games and simulations in pharmacy education has increased.

Pharmacy games are one such concept that intersect gamification and simulation, in which pharmacy student teams competitively manage simulated pharmacies, a concept included in the pharmacy curricula of seven international universities. Fens and colleagues collected data via a questionnaire completed by academic staff, and the collation of results of the same patient case was conducted at each university (April 2020 to March 2021) [3]. Universities reported positive experiences and student outcomes, suggesting that the intervention represents a potential tool to deliver capstone learning experiences, promote interprofessional education, reinforce patient safety, and prepare pharmacy graduates for future practice.

Hope and colleagues in Australia further analyzed the students’ written reflections of the pharmacy game using semantic thematic analysis [4]. The major themes that emerged were teamwork, patient-centeredness, medicines provision, future practice, and the learning experience. Student participants reported an intense and emotional experience in the gamified simulation, with many students revealing transformation in their skills, behaviors, and attitudes over its duration.

Kiles and colleagues report the choose your own adventure (CYOA) patient case format is an innovative activity that presents a patient case in an engaging way [5]. The CYOA patient case activity was designed by transforming a traditional paper patient case involving outpatient diabetes management into an interactive format utilizing an online platform, where students are sent down different routes depending on the choices they make. The CYOA activity improved self-reported knowledge of outpatient diabetes management and increased self-reported confidence in clinical decision-making skills.

Ryan and colleagues from Ireland describe the evaluation an integrated science and practice learning component in year one, using the World Health Organisation’s (WHO) analgesic ladder as a scaffold for case-based learning [6]. Highlights from an anonymous online student survey were the recognition by students of the importance of core science knowledge for practice, the enabling of integrated learning, and the suitability of the integrated component for entry-level.

Tommelein and colleagues from Belgium carried out an objective overview of nutritional topics discussed in community pharmacies to adapt the nutrition-related course content for pharmacy interns [7]. This study shows the importance of using real life practice examples in pharmacy education.

Of great interest during the pandemic, Falope and colleagues from US examined the role of the pharmacist in providing immunization services to pregnant women and concluded that pharmacists are in a good position to play a role in increasing immunization rates among pregnant women, and that pregnancy-specific content should be added to the immunization curricula [8].

Malekigorji from the UK describes a super blended teaching and learning model by hybridizing a classroom response system (CRS) with a flipped classroom (FC) and team-based learning (TBL) [9]. CRS allowed learners to use their smart devices (e.g., phones, tablets, and laptops) to respond to a variety of numerical, multiple-choice, short-answer, and open-ended questions posed during live classes and encouraged them to engage with classroom activities. This approach enabled educators to monitor student engagement throughout the year, facilitated formative assessment, and assisted teachers to create crude class performance prediction in summative assessment.

El Akel and colleagues from Lebanon focus on the opportunities and challenges encountered and present a model for experiential education in Lebanon [10]. Learning outcomes and thus students’ acquisition of predefined competencies are evaluated in actual practice settings through assessment tools. In Lebanon, the concept of an onsite preceptor is still new, and pharmacy practitioners still need to further develop their precepting skills. Yet, the dual-preceptorship concept that they describe allows them to overcome this limitation by depending heavily on the faculty preceptor to fill this gap.

Global health [11] and cultural competencies [12] are discussed in two papers from Schellhase and colleagues the US. The Global Health Experience Learning Progression (GHELP) model was derived to describe the process of student learning while on global health experiences. This progression model has three constructs and incorporates learning from external and internal influences. The model describes advancement from cultural awareness to cultural sensitivity and describes how student pharmacists develop global health knowledge, skills, and attitudes when participating in international experiential education. To prepare for an international advanced practice experience in clinical research, students competed a small preparatory module focused on intercultural learning and travel preparation. Students completed the Intercultural Development Inventory^®^ before the placement and four weeks after it. Some of the issues around using this tool are discussed in the paper.

Three papers discuss the development and evaluation of education for practicing pharmacists. Lorenzoni and colleagues developed “The Pharmaceutical Services and Access to Medicines Management Course” that has been adopted by 2500 pharmacists working in the public health system across Brazil [13]. A mixed methods evaluation identified important barriers to complete the course: high course workload, poor quality of Internet access, and a lack of support from the health services. Participants highlighted crucial features of the course that helped them develop key competencies: practical in-service activities, useful and realistic contents, and tutoring. These features helped participants overcome some important constraints described by them. The educational model described in this study was perceived as having a long-term impact on their behaviors and management practices in health services. Al Haqan describes the development of continuous professional development program for pharmacists in Kuwait [14]. A guiding competency framework and continuous feedback from program instructors added valuable support for pharmacists during the program and facilitated an impactful translation of education into practice. Terajima and colleagues explored perspectives on CE programs for foundation-level drugstore pharmacists in Japan [15]. They discovered that drugstore pharmacists in Japan have different continuing professional development requirements from community pharmacies. McGee and Colleagues in the US analysed survey data from targeted residency directors to preceptors [16]. A third of the programs required infectious diseases training as a mandatory rotation. Resident’s stewardship activities ranged from program to program; there was no consensus of the training activities, so the authors call for more standardised training activities to be developed by residence providers.

The number of pharmacy schools in Japan increased from 46 in 2002 to 74 in 2016. Takeda and Arakawa discuss the comparative results of the first cycle of the third-party accrediting organization, the Japan Accreditation Board for Pharmaceutical Education [17]. Private universities or schools tended to require more improvements in their pharmacy education than public ones in most of categories (10 out of 13 categories, and 3 significant differences found). These results suggest that new universities or schools established since 2003 have not yet established their own quality assurance mechanism within the institutions. The Japanese pharmacy education system or the assessment criteria need reviewing to bring about essential change.

We are grateful for all the colleagues who took the time to submit their publications while working through the pandemic. We originally asked for papers describing evolving North–South collaboration, South–South collaboration, and triangular collaborations to advance pharmacy education development to achieve universal health coverage, which is a global aim for the World Health Organization. These papers were lacking; a future addition should perhaps solely focus on this important area.

## References

[1] Mirzaian E., Franson K.L. (2021). Leading a Digital Transformation in Pharmacy Education with a Pandemic as the Accelerant. Pharmacy.

[2] Mak V., Sandhu A.K., Krishnan S. (2020). Using Simulation to Teach Methods for Improving Patient Literacy about Medicines. Pharmacy.

[3] Fens T., Hope D.L., Crawshaw S., Tommelein E., Dantuma-Wering C., Verdel B.M., Trečiokienė I., Solanki V., van Puijenbroek E.P., Taxis K. (2021). The International Pharmacy Game: A Comparison of Implementation in Seven Universities World-Wide. Pharmacy.

[4] Hope D.L., Rogers G.D., Grant G.D., King M.A. (2021). Experiential Learning in a Gamified Pharmacy Simulation: A Qualitative Exploration Guided by Semantic Analysis. Pharmacy.

[5] Kiles T.M., Hall E.A., Scott D., Cernasev A. (2021). Enhancing Student Knowledge of Diabetes through Virtual Choose Your Own Adventure Patient Case Format. Pharmacy.

[6] Ryan T.J., Ryder S.A., D’Arcy D.M., Quigley J.M., Ng N.N., Ong W.Q., Tey Z.H., O’Dwyer M., Walsh J.J. (2021). Development of Professional Attributes through Integration of Science and Practice at First-Year Pharmacy Level. Pharmacy.

[7] Tommelein E., De Boevre M., Vanhie L., Van Tongelen I., Boussery K., De Saeger S. (2021). Revisiting the Food- and Nutrition-Related Curriculum in Healthcare Education: An Example for Pharmacy Education. Pharmacy.

[8] Falope O., Vamos C., Izurieta R., Daley E., Kirby R.S. (2021). An Assessment of Pharmacy School Curricula in Florida and Inactivated Influenza Vaccine (IIV) Administration to Pregnant Women. Pharmacy.

[9] Malekigorji M., Hatahet T. (2020). Classroom Response System in a Super-Blended Learning and Teaching Model: Individual or Team-Based Learning?. Pharmacy.

[10] Akel M.E., Rahal M., Dabbous M., Mourad N., Dimassi A., Sakr F. (2021). Experiential Education in Pharmacy Curriculum: The Lebanese International University Model. Pharmacy.

[11] Schellhase E.M., Miller M.L., Malhotra J.V., Dascanio S.A., McLaughlin J.E., Steeb D.R. (2021). Development of a Global Health Learning Progression (GHELP) Model. Pharmacy.

[12] Schellhase E., Hasan I., Hendricks S., Miller M.L. (2021). Integration of Intercultural Learning into an International Advanced Pharmacy Practice Experience in London, England. Pharmacy.

[13] Lorenzoni A.A., Manzini F., da Trindade M.C.N., Storb B.H., Rech N., Farias M.R., Leite S.N. (2021). Attending a Blended In-Service Management Training in a Public Health System: Constraints and Opportunities for Pharmacists and Health Services. Pharmacy.

[14] Al-Haqan A., Al-Baghli S., Al-Enizi A.-B., Al-Dosari H., Waheedi S. (2020). The Development and Evaluation of a Structured Continuing Professional Development Programme for Pharmacists in Kuwait: A Feasibility Study. Pharmacy.

[15] Terajima T., Matsushita K., Yamada S., Suzuki H., Yano S., Makimura M., Yamamura S. (2020). Perspectives on Continuing Education Programs for Foundation-Level Drugstore Pharmacists in Japan. Pharmacy.

[16] McGee E.U., Mason-Callaway A.D., Rollins B.L. (2020). Are We Meeting the Demand for Pharmacist-Led Antimicrobial Stewardship Programs during Postgraduate Training-Year 1 (PGY1)?. Pharmacy.

[17] Takeda K., Arakawa N. (2021). A Comparative Exploration of Quality Assurance Results by the Third-Party Pharmaceutical Education Evaluation in Japan. Pharmacy.

